# Long-Term Oncological Outcomes Related to Lymphadenectomy in Clinical Stage I NSCLC: A Multicenter Retrospective Experience

**DOI:** 10.3390/curroncol32010031

**Published:** 2025-01-05

**Authors:** Beatrice Manfredini, Carmelina Cristina Zirafa, Alessandro Stefani, Gaetano Romano, Greta Alì, Riccardo Morganti, Ilaria Ceccarelli, Federico Davini, Pier Luigi Filosso, Franca Melfi

**Affiliations:** 1 Minimally Invasive and Robotic Thoracic Surgery, Surgical, Medical, Molecular and Critical Care Pathology Department, University of Pisa, 56126 Pisa, Italy; gaetano.romano@ao-pisa.toscana.it (G.R.); i.ceccarelli1@studenti.unipi.it (I.C.); f.davini@ao-pisa.toscana.it (F.D.); 2 Unit of Thoracic Surgery, Department of Medical and Surgical Sciences, University of Modena and Reggio Emilia, 41121 Modena, Italy; alessandro.stefani@unimore.it (A.S.); pierluigi.filosso@unimore.it (P.L.F.); 3 Pathological Anatomy, Surgical, Medical, Molecular and Critical Care Pathology Department, University Hospital of Pisa, 56126 Pisa, Italy; greta.ali@unipi.it; 4 Section of Statistics, University Hospital of Pisa, 56126 Pisa, Italy; r.morganti@ao-pisa.toscana.it; 5 Unit of Thoracic Surgery, Department of Pharmacy and Health and Nutrition Sciences, University of Calabria, 87036 Rende, Italy; franca.melfi@unipi.it

**Keywords:** early stage, clinical stage I, NSCLC, lymphadenectomy

## Abstract

Background: Lymphadenectomy is considered a key part of the radical treatment of resectable lung cancer, although its appropriate extension in early stages is a debated topic due to the great heterogeneity of studies in the literature. This study aims to evaluate the impact of lymphadenectomy extent on survival and recurrence in the treatment of early-stage NSCLC patients undergoing lobectomy and lymph node dissection. Methods: Data from clinical stage I NSCLC patients undergoing lobectomy and hilar-mediastinal lymphadenectomy at two thoracic surgery centers from 2016 to 2019 were retrospectively evaluated. Information regarding perioperative outcomes and lymphadenectomy details was collected and analyzed, and their impact on OS, CSS, and DFS was assessed. Results: During the period under review, 323 patients with stage cI lung cancer underwent lobectomy with lymphadenectomy. Statistical analysis showed that the evaluated lymph nodal factors (mean number of lymph nodes removed and number and type of lymph node station explored) did not statistically significantly impact OS, CSS, and DFS at a median follow-up of 59 months (IQR 45–71). Conclusions: The results of this study suggest that a less invasive procedure than systematic lymphadenectomy could be performed in early-stage cases with adequate preoperative staging.

## 1. Introduction

In recent decades, because of the high incidence of lung cancer, screening programs targeting high-risk patients have been proposed, leading to an increase in the detection of early stages of non-small cell lung cancer (NSCLC), partly due to improved instrumental investigations for diagnosis and staging [1].

In the surgical treatment of lung malignancy, an adequate lymphadenectomy is essential to achieve accurate staging and, consequently, to identify patients who may benefit from adjuvant therapy [2]. However, the extent of lymphadenectomy in patients with early-stage NSCLC is a controversial topic to date [3,4,5,6].

The greatest current critical issue is the heterogeneity of studies present in the literature on this topic, including the most recent ones, in terms of disease stage and lymphadenectomy technique, making it difficult to compare results and obtain reliable and reproducible conclusions [7,8,9]. Many of the studies, being dated, also do not include a whole-body positron emission tomography scan (18-FDG PET-CT) in the preoperative staging [10,11,12,13,14,15], leading to incorrect identification of early clinical stages following the inclusion of advanced stages. Furthermore, lymph node dissection, surgical techniques, and nomenclature are often not standardized across the different published trials, and details on the number of lymph nodes removed at different sites are lacking [11], making it difficult to relate the results of different studies [16].

Consequently, the role of lymphadenectomy in early-stage NSCLC still seems to be a topic of debate.

This study aimed to evaluate the effect of lymphadenectomy in the treatment of clinical stage I NSCLC, using reproducible factors related to lymphadenectomy to obtain results that can guide clinical practice. Specifically, this is a preliminary study to establish from the data on surgical lymphadenectomy performed by the participating centers whether this procedure has an impact on survival and recurrence in terms of overall survival (OS), cancer-specific survival (CSS), and disease-free survival (DFS) in order to define the criteria for lymphadenectomy in future studies.

## 2. Materials and Methods

Data from all patients who underwent pulmonary lobectomy and hilar-mediastinal lymphadenectomy with curative intent, from January 2016 to December 2019, at the Unit of Minimally Invasive and Robotic Thoracic Surgery of the University Hospital of Pisa and the Unit of Thoracic Surgery of the University Hospital of Modena and Reggio Emilia were retrospectively collected and analyzed. We only included patients who underwent pulmonary lobectomy to have a homogeneous sample.

The following were the inclusion criteria:

(1) Diagnosis of NSCLC;
(2) Clinical stage I;
(3) The execution of lymphadenectomy including at least one mediastinal lymph node station;
(4) Number of lymph nodes removed and corresponding lymph node stations available on definitive histological examination.

Surgical procedures were performed with open surgery by lateral or postero-lateral thoracotomy, three-access videothoracoscopic approach, or fully endoscopic robotic approach with four ports, using the da Vinci Xi^®^ robotic system (Intuitive, Sunnyvale, CA, USA).

The patients’ demographic and clinicopathological characteristics and intra- and post-operative findings were collected.

Comorbidities were assessed according to the Charlson–Deyo score [17].

The clinical stage was based on information obtained by total-body computed tomography (CT) with contrast enhancement and 18-FDG PET-CT, according to the eighth edition of the TNM staging system for lung cancer [18].

In the case of central neoplasms or doubtful lymph node involvement on preoperative imaging (enlarged lymph nodes on CT scan or positive on 18-FDG PET-CT), only patients who tested negative on lymph node sampling by EBUS or mediastinoscopy were enrolled in the study. In each case of suspected lymph node metastatic involvement by macroscopic pathological appearance, intraoperative histological examination was performed, and patients confirmed as N0 intraoperatively were enrolled.

Upstaging was assessed as an increase in stage category on the final histological examination (e.g., stage I to stage II; stage II to stage III). The upstaging of the N parameter was classified in the case of pathological N1 or N2 compared to clinical N0.

Preoperatively, all patients underwent respiratory functional assessment, cardiological evaluation, blood tests, and anesthesiological evaluation to define the American Society of Anesthesiologists’ Classification of Physical Health (ASA) risk class.

Post-operative complications were analyzed using the Clavien–Dindo Classification of Complications (CDCC) [19].

Perioperative mortality was assessed as any death occurring within the first 30 days after surgery.

Regarding lymphadenectomy, the total number of lymph nodes removed, and the number and location of hilar and mediastinal lymph node stations explored were analyzed. The number of lymph node stations refers to the number of stations examined during surgery, whereas the number of lymph nodes is the addition of the lymph nodes removed from the same station and counted by the pathologist.

In addition, for lymph node station N1, we referred to lymph node stations 10, 11, and 12, which were removed during lymphadenectomy. For mediastinal stations 2 to 9, we referred to lymph node station N2.

After obtaining the data concerning the lymphadenectomy performed during surgery, we went on to evaluate the impact of the same on OS, CSS, and DFS.

OS was defined as the time in months from disease diagnosis to death from each cause.

CSS was defined as the time in months from diagnosis of disease to death from lung malignancy.

DFS was defined as the time in months from disease diagnosis to recurrence or death.

### 2.1. Follow-Up

All patients underwent objective examination and contrast-enhanced chest–abdomen CT scan every six months for the first two years after surgery and then once a year, in line with the international guidelines [20]. In case of suspicion of disease recurrence, patients underwent 18-FDG PET-CT, brain MRI, and CT-guided or bronchoscopic biopsy, based on medical evaluation.

### 2.2. Statistical Analysis

Regarding statistical investigations, categorical data were described by absolute or relative frequency (%), and continuous data were summarized with mean (standard deviation and range) or median (range OR interquartile range IQR). Curves of OS, CSS, and DFS were calculated using the Kaplan–Meier method and to analyze the influence of the lymph node factors on the survival univariate, Cox regression and competing-risks regression were performed. Significance was set at 0.05, and all analyses were carried out with SPSS v.29 technology.

## 3. Results

From January 2016 to December 2019, 323 patients (149 men 46%; 174 women 54%; median age 69 years; range 32–84 years) underwent pulmonary lobectomy with lymphadenectomy for clinical stage I NSCLC (Table 1).

Preoperative spirometry showed a median FEV1 value of 2.19 L (range 0.79–4.74 L) and a median FEV1/FVC ratio of 75% (range 34–125%).

The median Charlson–Deyo comorbidity score was 5 (range 1–11), with a percentage of patients with an ASA risk class > 2 of 51% (164 patients) (Table 1).

The most frequent clinical stage was IA2 (170 patients; 53%).

The anatomical distribution of the lobectomies performed is shown in Table 2.

Regarding the surgical technique, 142 (44%) patients were treated with robotic surgery, 119 (37%) with open surgery, and 62 (18%) using the videothoracoscopic approach.

The median surgical time was 180 min (range 50–535 min), and the median length of hospital stay was 5 days (range 2–24). Intraoperative complications were linked in most cases to arterial bleeding (six cases) and in one case to arrhythmia with hemodynamic instability. The post-operative complication rate was 25% (82 patients). Most of the complications that occurred were minor (38 grade I according to the CDCC classification, 12%). The most frequent post-operative complication was prolonged air leak (37 patients, 11%). Two patients died within 30 days after surgery (0.6%), in one case due to respiratory failure caused by the exacerbation of the underlying interstitial lung disease and in one case because of cerebrovascular accident (Table 2).

In most of the cases, pathological examination diagnosed an adenocarcinoma (236 patients, 73%), while the most common pathological stage was IA2 (128 patients, 40%).

An upstaging was observed in 24 patients (7.4%), all due to upstaging of the N parameter and one upstaging also of the T parameter (Table 2). In detail, occult lymph node metastases were found in hilar lymph node stations (N1) in 18 (6%) patients and in mediastinal lymph node stations (N2) in 6 (2%) patients.

After evaluation by the multidisciplinary lung cancer tumor board based on the tumor characteristics and the clinical condition, 5 (2%) patients of the 18 patients with pathologically staged IIB and 4 (1%) of the 6 patients with pathologically staged IIIA received adjuvant chemotherapy.

The average number of lymph nodes removed was 13 (SD 6, range 1–35). The exploration of at least three mediastinal lymph node stations was observed in 66% of the cases; whereas a single mediastinal station was removed in 12% of the patients (Table 3).

Table 4 shows the distribution of hilar lymph node stations removed by the surgeon during lymphadenectomy.

The mediastinal station most frequently harvested on the right was station 4 (119 patients; 62%) and station 5 on the left (95 patients, 29%) (Table 5).

**Figure 1:**
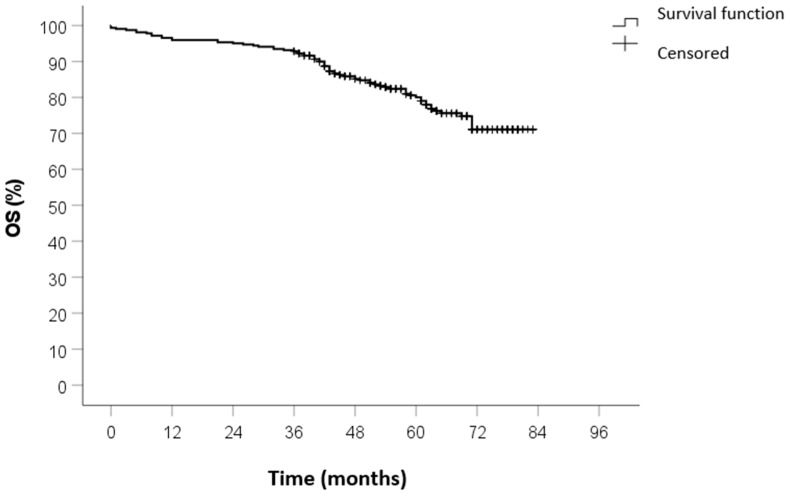
OS survival curve calculated by Kaplan–Meier method.

**Figure 2:**
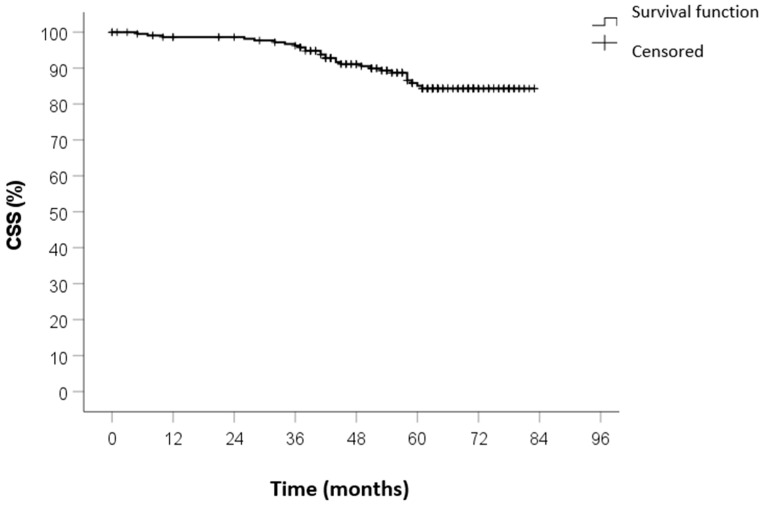
CSS survival curve calculated by Kaplan–Meier method.

**Figure 3:**
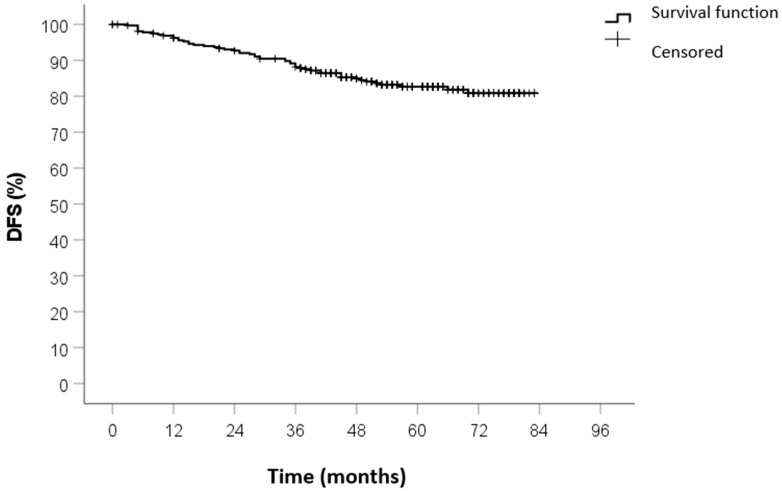
DFS survival curve calculated by Kaplan–Meier method.

No patients were lost to follow-up. The median duration of follow-up was 59 months (IQR 45–71). The 5-year OS was 82%, the 5-year CSS was 88%, and the 5-year DFS was 84%. The Kaplan–Meier survival curves of OS, CSS, and DFS are shown in Figure 1, Figure 2 and Figure 3.

The impact of the following factors related to lymphadenectomy on survival and recurrence was analyzed: mean number of lymph nodes removed; mean number of N2 lymph node stations harvested; mean number of N1 lymph node stations harvested; three or more mediastinal lymph node stations harvested; one mediastinal lymph node station harvested.

Statistical analysis showed that none of the lymphadenectomy-related factors considered had a statistically significant effect on OS, DFS, and CSS (Table 6 and Table 7).

## 4. Discussion

The treatment of choice associated with better prognosis in resectable NSCLC is pulmonary lobectomy with hilar-mediastinal lymphadenectomy [4]. Lymph node dissection is a crucial step in the surgical treatment of lung cancer to obtain an accurate staging, then assess the prognosis, and plan multimodal treatment when necessary [2,21,22]. However, the international guidelines differ in terms of procedural indications and nomenclature of lymphadenectomy.

The ESTS guidelines on lymph node staging, published in 2006, recommend systematic lymphadenectomy in all cases of resectable lung cancer, with the removal of at least three mediastinal lymph node stations. Conversely, lymph node sampling should be reserved for high-risk patients, and lobe-specific lymphadenectomy is indicated for peripheral T1 squamous cell carcinoma with negative hilar and interlobar lymph nodes [3]. The NCCN guidelines advise that complete resection requires dissection or systematic sampling of lymph nodes and the highest tumor-negative mediastinal lymph node. They specify that resection and mapping of N1 and N2 lymph nodes should be a routine component of lung cancer resections, with sampling of at least one N1 and three N2 stations or complete lymph node dissection [4]. Finally, for the treatment of early-stage NSCLC, ESMO guidelines [5] recommend systematic lymphadenectomy, in agreement with the IASCL [12], which defines it as the removal of at least six lymph nodes/stations, three of which should be mediastinal, including the sub-carinal station, with no metastases found in most cranial contexts [6].

Even today, there is controversy about the extent of lymphadenectomy in early-stage NSCLC, and as already mentioned, there is no uniformity in the definition of the different types of lymph node dissection, creating confusion in the practical application of international recommendations.

The first prospective randomized controlled trial on the comparison of systematic lymphadenectomy and lymph node sampling in the treatment of resectable NSCLC (stage cI-IIIA) was published in 1998 [14] and concluded that complete lymphadenectomy is not necessary in patients with clinical N0, as it does not impact long-term oncological outcomes.

In the following years, several trials comparing different types of lymphadenectomy were published with discordant results. Wu et al. conducted a prospective randomized trial that demonstrated improvement in survival and recurrence-free survival after systematic lymphadenectomy in the treatment of clinical stage I–IIIA NSCLC when compared with lymph node sampling [23]. In contrast, in 2011, Darling et al. [24] showed that systematic lymphadenectomy does not increase survival in early-stage NSCLC (pT1-T2N0-N1M0) in case of negative hilar and mediastinal systematic sampling. In 2023, Huang et al. [25], in a retrospective study of the treatment of clinical stage I NSCLC with lobectomy, showed that lobe-specific and systemic lymphadenectomy did not differ in long-term oncological outcomes in terms of OS, CSS, and DFS.

As previously mentioned, the published studies resulted in inconclusive and non-comparable results due to the heterogeneity of the sample in terms of stage and lymphadenectomy details. Additionally, the clinical staging accuracy is limited, as it was based solely on CT investigations prior to the introduction of 18-FDG PET-CT in the preoperative evaluation. [10,11,12,13,14,15]. Consequently, one of the most recent systematic reviews and meta-analyses of randomized controlled trials comparing systematic lymphadenectomy with mediastinal lymph node sampling led to uncertain conclusions due to a high risk of bias [7].

To obtain definite results that can guide clinical practice, it is necessary to homogenize the study population. Many studies have included a wide range of disease stages, leading to questionable results [14,23]. Furthermore, defining the parameters of lymphadenectomy, such as the number of lymph nodes removed and the corresponding lymph node stations, appears crucial to allow reproducibility in subsequent studies.

In this study, only clinical stage I NSCLC patients undergoing pulmonary lobectomy and hilar-mediastinal lymphadenectomy were included to reduce the risk of possible confounding factors. Patient selection is the strength of this report, as all included patients underwent 18-FDG PET-CT, reducing the risk of misdiagnosed lymph nodes or distant metastases by investigating suspected cases pre- or intra-operatively.

According to our results, none of the lymphadenectomy factors analyzed have a statistically significant impact on OS, CSS, and DFS. It can be reasonably deduced that the implementation of an adequate preoperative clinical staging system, which accurately identifies patients at an early clinical stage, may result in the possibility of performing a less invasive procedure than systematic mediastinal lymphadenectomy. This would have the additional benefit of avoiding a procedure with a higher morbidity and mortality rate as well as a longer operative time for a presumed local stage of disease. Meta-analyses and retrospective studies have indeed shown that complete systematic lymphadenectomy involves more perioperative complications than less extensive forms of lymph node dissection [9,13,26].

In NSCLC, upstaging is considered a factor related to the quality of lymphadenectomy, identifying those patients who may benefit from adjuvant therapies and improving prognosis [27,28]. The N-factor-related upstaging rate is relatively low in this study despite the positive survival and recurrence data, emphasizing that occult lymph node metastases found by standard histopathological methods are infrequent in early stages in the presence of adequate preoperative staging. Darling et al. [24] and Veronesi et al. [29] also reported a low N upstaging rate (4% and 6.2%, respectively) in a selected patient population, demonstrating that the risk of lymph node involvement is reduced in these cases.

In this study, 16.7% of patients presented recurrence. Specifically, 15 (5%) patients had local recurrence, 22 (8%) patients had distant recurrence, and 17 (5%) patients had both local and distant recurrence. This could be explained by the presence of occult lymph node micrometastases, which is a marker of primary malignancy with high metastatic potential. Currently, there are several molecular biology methods available for the detection of micrometastases in NSCLC, such as polymerase chain reaction (PCR), immunohistochemistry (IHC), and one-step nucleic acid amplification (OSNA) [30].

There is a need to direct research towards the identification of increasingly specific and sensitive markers of micrometastasis to achieve more accurate staging and to define patients who might benefit from more aggressive lymphadenectomy. Tsai et al. [31] found in their retrospective study that abnormal serum levels of carcinoembryonic antigen, solid tumor diameter ≥ 1.3 cm, and consolidation to tumor ratio ≥ 0.50 on chest CT were predictive factors for lymph node upstaging in patients with clinical T1a-bN0M0 adenocarcinoma undergoing lobectomy and systematic lymphadenectomy, suggesting that if none of these predictive factors is positive, a less invasive procedure with lymph node sampling or no lymphadenectomy may be a reasonable alternative.

The limitations of this study are its retrospective nature and the participation of only two centers. Another limitation of this study is that lymph nodes were not removed in their entirety but in fragments, especially with the videothoracoscopic technique, which may have introduced bias.

## 5. Conclusions

The impact of lymphadenectomy on survival and recurrence in early clinical stage NSCLC remains controversial.

This study showed that the extent of mediastinal lymphadenectomy (number of lymph nodes removed and number and type of lymph node stations harvested) does not influence OS, CSS, and DFS in clinical stage I NSCLC patients.

Accurate preoperative and intraoperative staging is essential to identify clinical stage I patients and, consequently, those for whom performing a less invasive procedure than systematic mediastinal lymphadenectomy might be reasonable.

## Figures and Tables

**Table 1:** Population characteristics. Statistics: median (range) or absolute frequency (%).

Characteristics	Statistics
Total number of patients	323
Men	149 (46)
Women	174 (54)
Age (years)	69 (32–84)
Smoke	258 (80)
Yes	105 (33)
Ex	153 (47)
No	65 (20)
Charlson–Deyo comorbidity score Comorbidity	5 (1–11)
Presence	303 (94)
Other neoplasms	92 (28)
Respiratory disease	83 (26)
Cardiovascular disease	124 (38)
Kidney disease	10 (3)
Liver disease	10 (3)
Hypertension	148 (46)
Diabetes mellitus II	35 (11)
ASA Risk Class	
I	9 (3)
II	150 (46)
III	157 (49)
IV	7 (2)
FEV1 (liters)	2.19 (0.79–4.74)
FEV1/FVC (%)	75 (34–125)
BMI	25.9 (15.6–42.7)
PET-TC 18-FDG SUV max lesion	4.5 (1–23)
cStage	
IA1	30 (9)
IA2	170 (53)
IA3	90 (28)
IB	33 (10)

**Table 2:** Intra- and post-operative results. Statistics: median (range) or absolute frequency (%).

Characteristics	Statistics
Surgical technique	
Open	119 (37)
Robot	142 (44)
VATS	62 (19)
Type of lobectomy	
Upper left	77 (24)
Lower left	34 (11)
Upper right	126 (39)
Middle	26 (8)
Lower right	60 (19)
Surgical time (min)	180 (50–535)
Intraoperative complications	7 (2)
Hospital stay (days)	5 (2–24)
Chest-tube duration (days)	3 (1–36)
30-day mortality	2 (0.6)
Post-operative complications	82 (25)
Prolonged air leak	37 (11)
Anemization with transfusion	12 (4)
Atrial fibrillation	14 (4)
CDCC complication grade	
I	38 (12)
II	32 (10)
III	12 (4)
Histology	
Adenocarcinoma	236 (73)
Squamous carcinoma	47 (16)
Other	40 (12)
pStage	
IA1	23 (7)
IA2	128 (40)
IA3	62 (19)
IB	86 (27)
IIB	18 (6)
IIIA	6 (2)
General upstaging	24 (7.4)
Upstaging N	23 (7.1)
Open	9 (3)
Robot	9 (3)
VATS	5 (2)
N1	18 (6)
N2	6 (2)

**Table 3:** Factors related to lymphadenectomy. Statistics: mean (SD; range) or absolute frequency (%).

Factors	Statistics
Mean number of lymph nodes	13 (6; 1–35)
Mean number of lymph node stations	5 (1.3; 2–9)
Mean number of N2 lymph node stations	3 (1.1; 1–6)
Mean number of N1 lymph node stations	2 (0.6; 1–3)
N2 stations ≥ 3	
No	110 (34)
Yes	213 (66)
1 N2 station	
No	283 (88)
Yes	40 (12)

**Table 4:** Hilar lymph node stations removed. Statistics: frequency (%).

	Total
10	303 (94)
11	239 (74)
12	53 (16)

**Table 5:** Mediastinal lymph node stations removed. Statistics: frequency (%).

	Right	Left
2	56 (17)	0 (0)
3	41 (13)	0 (0)
4	199 (62)	13 (4)
5	0 (0)	95 (29)
6	0 (0)	49 (15)
7	172 (53)	67 (21)
8	69 (21)	12 (4)
9	77 (24)	67 (21)

**Table 6:** Survival factor analysis of lymphadenectomy. RC: regression coefficient.

	RC	HR (95% CI)	*p*-Value
Lymph node factor OS analysis			
Number of lymph nodes	0.018	1.018 (0.979–1.059)	0.363
Number of lymph node stations	0.036	1.037 (0.861–1.249)	0.702
Number of N2 lymph node stations	−0.005	0.995 (0.801–1.236)	0.965
Number of N1 lymph node stations	0.192	1.212 (0.817–1.798)	0.340
N2 stations ≥ 3	−0.165	0.848 (0.527–1.365)	0.497
1 N2 station	−0.111	0.895 (0.444–1.803)	0.756
Lymph node factor DFS analysis			
Number of lymph nodes	0.002	1.002 (0.956–1.050)	0.940
Number of lymph node stations	0.024	1.024 (0.828–1.266)	0.825
Number of N2 lymph node stations	−0.007	0.993 (0.772–1.276)	0.955
Number of N1 lymph node stations	0.141	1.152 (0.735–1.804)	0.538
N2 stations ≥ 3	0.148	1.160 (0.735–1.804)	0.615
1 N2 station	0.034	1.034 (0.467–2.292)	0.934

**Table 7:** Analysis of lymphadenectomy factors influencing CSS using competing-risks regression.

	RC	HR	Standard Error	*p*-Value
Lymph node factor CSS analysis				
Number of lymph nodes	−0.025	0.975	0.039	0.520
Number of lymph node stations	0.078	1.081	0.152	0.610
Number of N2 lymph node stations	−0.068	0.934	0.156	0.660
Number of N1 lymph node stations	0.581	1.788	0.348	0.095
N2 stations ≥ 3	−0.027	0.974	0.391	0.950
1 N2 station	−0.263	0.769	0.617	0.670

## Data Availability

The data underlying this article will be shared by the corresponding author upon reasonable request.
